# Smear layer removal efficacy of different irrigation techniques in conservatively instrumented root canals

**DOI:** 10.1016/j.jds.2024.01.008

**Published:** 2024-01-24

**Authors:** Sota Mochizuki, Satoshi Watanabe, Jiayi Liu, Takashi Okiji

**Affiliations:** Department of Pulp Biology and Endodontics, Division of Oral Health Sciences, Graduate School of Medical and Dental Sciences, Tokyo Medical and Dental University (TMDU), Tokyo, Japan

**Keywords:** ErbiumYAG lasers, Er-YAG laser, Er-YAG lasers, Instrumentation, Root canal irrigants, Root canal therapy

## Abstract

**Background/purpose:**

Establishing effective irrigation methods is warranted to ensure the predictability of minimally invasive root canal instrumentation. This study aimed to compare the smear layer removal efficacy of different irrigation techniques in root canals instrumented with TruNatomy nickel-titanium rotary instruments.

**Materials and methods:**

Experiment 1: Extracted human mandibular incisors were instrumented using TruNatomy Shaping Files up to Small (#20/0.04), Prime (#26/0.04), or Medium (#36/0.03) (n = 17, each) and irrigated with EDTA, NaOCl, and again with EDTA using syringe irrigation (SI). Experiment 2: Extracted mandibular incisors were instrumented using TruNatomy Small and irrigated with EDTA and NaOCl as in Experiment 1, using (1) conventional laser-activated irrigation (LAI) with an erbium-doped yttrium aluminum garnet laser unit at 30 mJ/10 Hz (LAI 30/10) or 70 mJ/20 Hz (LAI 70/20), (2) photon-induced photoacoustic streaming (PIPS) (20 mJ/15 Hz), (3) ultrasonic-activated irrigation, or (4) SI (n = 13 each). Five additional canals served as negative controls (no irrigation). The smear layer on the canal wall was scored under scanning electron microscopy.

**Results:**

In Experiment 1, the TruNatomy Small group exhibited significantly higher smear layer scores compared to the other groups in the apical and middle thirds. In Experiment 2, the LAI 70/20 and PIPS groups demonstrated significantly lower smear layer scores compared to the LAI 30/10 and SI groups in the apical third.

**Conclusion:**

Conservative instrumentation using the TruNatomy Small reduced the cleaning efficacy of irrigation. However, PIPS performed satisfactory for smear layer removal without injury to the root canal wall.

## Introduction

Mechanical instrumentation is crucial for efficient removal of pathogens from the root canal system.^1^^,^^2^ There is a great deal of interest in minimally invasive root canal instrumentation,^3^, ^4^, ^5^ which involves preservation of the cervical dentin to reduce the risk of tooth fracture.^6^^,^^7^ TruNatomy (Dentsply Sirona, Ballaigues, Switzerland) is a recently introduced heat-treated nickel-titanium rotary system designed based on the concept of minimally invasive instrumentation; it is characterized by a regressive taper and a smaller maximum flute diameter of 0.8 mm. Furthermore, TruNatomy incorporates an off-centered parallelogram cross-section, a unique design element purported to significantly enhance flexibility. This feature is instrumental in promoting minimal invasiveness and in ensuring more effective preservation of dentin.^8^^,^^9^ However, concern exists regarding the disinfection efficacy of minimally invasive instrumentation because the use of such devices may compromise the effective removal of intracanal pathogens,^10^, ^11^, ^12^ due particularly to insufficient irrigant penetration with conventional syringe irrigation (SI).^13^ Thus, facilitating irrigant flow using irrigant activation techniques seems mandatory to ensure the predictability of minimally invasive root canal instrumentation.

Several irrigant activation techniques have been employed, including sonic-activated irrigation, ultrasonic-activated irrigation (UAI), and laser-activated irrigation (LAI). UAI involves acoustic streaming and cavitation, which may improve the flushing of irrigants in inaccessible canal areas.^14^^,^^15^ LAI using an erbium-doped yttrium aluminum garnet (Er:YAG) laser generates high fluid flow through rapid bubble growth and collapse, thus creating a fluid movement with secondary cavitation effects in the canal space and enhancing canal debridement.^16^, ^17^, ^18^

The efficacy of LAI has been demonstrated in cleaning complex canal anatomy, including the isthmus,^19^^,^^20^ lateral canals,^21^ apical areas beyond canal ledges^22^ and fractured instruments.^23^ Photon-induced photoacoustic streaming (PIPS), which uses an Er:YAG laser at 2940 nm (LightWalker; Fotona, Ljubljana, Slovenia), is an effective LAI technique that involves laser emission at low energy levels and a short pulse duration using a conical laser tip. The cleaning effect of PIPS reportedly extends to the apical area despite irradiation from the pulp chamber or canal orifice.^24^^,^^25^

Establishing effective irrigation methods is still warranted to solve the difficulty of thorough cleaning in the apical area of minimally instrumented root canals.^13^ Also, there have been no reports on the efficacy of different irrigation techniques in cleaning root canals conservatively instrumented with the TruNatomy system. Therefore, this study was performed to compare different irrigation techniques in terms of the smear layer removal efficacy from canals instrumented with TruNatomy instruments. The null hypothesis was that there is no difference in the smear layer removal efficacy among (1) final instrumentation sizes and (2) irrigation techniques.

## Materials and methods

### Tooth samples

Extracted human mandibular incisors were used with approval by the Institutional Review Board of our institution (No. D2014-033). The patients were informed, at the time of extraction, that the extracted teeth would be used for research purposes, and written approval was obtained from all the subjects.

The sample sizes needed to identify significant differences were calculated using G∗Power software version 3.1.9 (Heinrich-Heine Universität, Düsseldorf, Germany). According to a previous study,^21^ a sample size of 17 per group for Experiment 1 and 13 per group for Experiment 2 was obtained by setting an effect size of 0.5, a significance level of 0.05, and a power of 95 %. The selected teeth had mature roots without caries, cracks, endodontic treatment, or restorations; a root length (cementoenamel junction to apex) of ≥13 mm; and radiographic features of a narrow canal with a curvature of <10° according to Schneider's method.^26^

### Root canal instrumentation

Following access opening, the teeth that allowed the introduction of a #25 K-file (Zipperer, Munich, Germany) to half the root length were excluded. Apical patency was confirmed using a #10 K-file barely visible at the apical foramen. The working length (WL) was set to 18 mm from the point at which the file was withdrawn 1 mm from the apical foramen. An orifice-shaping instrument (TruNatomy Orifice Modifier; Dentsply Sirona, Ballaigues, Switzerland) was used to 9 mm from the WL. To establish a closed system, the root was placed in a hollow plastic tube filled with silicone rubber impression material (Exafine; GC, Tokyo, Japan).

The teeth were instrumented to the WL with TruNatomy Glider (#17/0.02) followed by TruNatomy Shaping File(s) up to either Small (#20/0.04 regressive taper), Prime (#26/0.04 regressive taper), or Medium (#36/0.03 regressive taper) using an endodontic motor (Tri Auto ZX2; J. Morita, Kyoto, Japan) in accordance with the manufacturer's recommendations (500 rpm/1.5 N cm). During instrumentation, the canals were filled with a lubricant (RC-Prep; Premier Dental, Philadelphia, PA, USA). After each instrument change, the canals were irrigated with 1 mL distilled water to avoid neutralizing the effect of RC-Prep, and canal patency was manually confirmed using a #10 K-file.

### Irrigation procedures

Post-instrumentation irrigation was conducted according to the protocol described in Mancini et al.^27^ with slight modifications (Table 1). The protocol involved an alternate use of 14.3 % ethylenediaminetetraacetic acid (EDTA) (Morhonine; GC Showa Yakuhin, Tokyo, Japan) and 6 % sodium hypochlorite (NaOCl) (Dental Antiformin; Nippon Shika Yakuhin, Shimonoseki, Japan), both of which were delivered with a 3.0 mL-syringe (Nipro, Osaka, Japan) with a 30-G Luer-Lok polypropylene irrigation needle (TruNatomy Irrigation Needle, 0.025 taper; Dentsply Sirona), and activated with various activation techniques as described below. Irrigant activation was not conducted in teeth in which only SI was performed.

**Table 1:** Irrigation protocol.

	Irrigant	Activation^a^
1	14.3 % EDTA (3 mL)	30 s
2	distilled water (3 mL)	30 s
3	6 % NaOCl (3 mL)	30 s
4	resting time (30 s)^b^	
5	6 % NaOCl (3 mL)	30 s
6	resting time (30 s)^b^	
7	distilled water (3 mL)	30 s
8	14.3 % EDTA (3 mL)	30 s
9	distilled water (3 mL)	30 s

a No activation in canals in which only syringe irrigation was performed.

b Canals were filled with NaOCl without activation. EDTA, ethylenediaminetetraacetic acid; NaOCl, sodium hypochlorite.

In Experiment 1, the effect of final instrumentation size on irrigation efficacy was examined. Fifty-one teeth were randomly assigned to three groups in which instrumentation was performed up to TruNatomy Small, Prime, or Medium (n = 17 each). The specimens underwent SI with the 30-G needle inserted 1 mm short of its binding position at a flow rate of 6 mL/min. The irrigation was conducted as in Table 1 without irrigant activation.

Experiment 2 aimed to compare the efficacy of active irrigation protocols in minimally instrumented canals. Sixty-five teeth were instrumented up to TruNatomy Small and randomly assigned to 5 groups (n = 13 each) as follows:

1. LAI 30/10 group: A 2940-nm Er:YAG laser (ErwinAdvErL; Morita Manufacturing, Kyoto, Japan) equipped with a conical 200-μm-diameter fiber tip (R200T; Morita Manufacturing) was used. The laser fiber tip was inserted 5 mm short of the WL without touching the canal wall. The pulse energy, frequency, and pulse duration were 30 mJ, 10 Hz, and 300 μs, respectively.
2. LAI 70/20 group: The settings were the same as those in the LAI 30/10 group, except the pulse energy was set at 70 mJ and frequency was set at 20 Hz.
3. PIPS group: A 2940-nm Er:YAG laser (LightWalker; Fotona, Ljubljana, Slovenia) equipped with a conical 400-μm-diameter quartz tip (PIPS 400/14 tip; Fotona) was used. The tip was placed at the root canal orifice (15 mm short of the WL). The pulse energy, frequency, and pulse duration were 20 mJ, 15 Hz, and 50 μs, respectively.
4. UAI group: A #10/0.02 stainless steel tip (U file; Pierce, Tokyo, Japan) was connected to an ultrasonic unit (ENAC 11W; Osada, Tokyo, Japan), placed 1 mm short of the WL without touching the canal wall and activated at a power setting of 3 (highest setting recommended by the manufacturer) at 30 kHz and 3.6 W.
5. SI group: The same irrigation procedure was used as in Experiment 1.

Five additional teeth served as negative controls in which the canal was instrumented but no irrigation was conducted.

Irrigation was conducted according to the protocol shown in Table 1 (no activation in the SI group). Irrigants were continuously delivered to the pulp chamber with the 30-G needle and syringe used for SI while the irrigant activation was conducted for every activation technique.

### Scanning electron microscopy

The roots were carefully split with a chisel after preparing longitudinal grooves on the mesial and distal surfaces using a diamond disc without penetration into the canals. A properly fitting gutta-percha cone (TruNatomy Conform Fit Gutta-Percha Points; Dentsply Sirona) was placed in the canal to limit contamination by dentin fragments. Half of each sample was randomly chosen and marked with a razor blade every 1.0 mm from WL -1.0 to −12.0 mm under a stereomicroscope. The samples were then dehydrated, desiccated, platinum-coated, and examined under scanning electron microscopy (SEM) (JSM-7900F; JEOL, Tokyo, Japan) at 15 kV.

**Figure 1:**
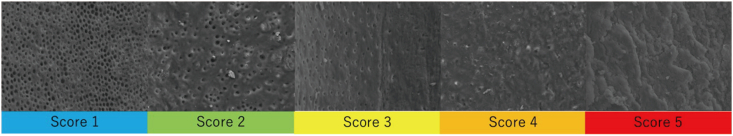
Smear layer scoring system according to Hülsmann M et al.^28^ and representative images of each score. Score 1, no smear layer, dentinal tubuli open; Score 2, small amount of smear layer, some dentinal tubuli open; Score 3, homogenous smear layer covering the root canal wall, only few dentinal tubuli open; Score 4, complete root canal wall covered by a homogenous smear layer, no open dentinal tubuli; Score 5, heavy, nonhomogenous smear layer covering the complete root canal wall.

Following overview inspection (30 × ), the samples were observed at 200 × magnification to ensure the position of the markings. An image at 1000 × magnification was taken from the center of the field of view within each area between neighboring markings, yielding 12 images per tooth. The images were grouped as apical third (1.0–4.0 mm short of the WL), middle third (5.0–8.0 mm short of the WL), and coronal third (9.0–12.0 mm short of the WL) (4 images in each third). Two calibrated and blinded observers evaluated the 840 photographs for the smear layer on the canal wall using a 5-score index system^28^ (Fig. 1).

### Statistical analysis

Normality was tested with the Kolmogorov-Smirnov test. Data were analyzed using the Kruskal–Wallis test and Mann–Whitney U test with Bonferroni correction. Interobserver and intraobserver agreement was analyzed using Cohen's kappa test. All analyses were performed using SPSS, version 28.0.1.0 (IBM, Armonk, NY, USA), and differences were considered significant at *P* < 0.05.

## Results

In Experiment 1, the kappa values were 0.78 for interobserver agreement and 0.77 and 0.79 for intraobserver agreement (substantial agreement). In Experiment 2, an interobserver kappa value of 0.83 and intraobserver kappa values of 0.81 and 0.82 were obtained (almost perfect agreement).

### Experiment 1

**Figure 2:**
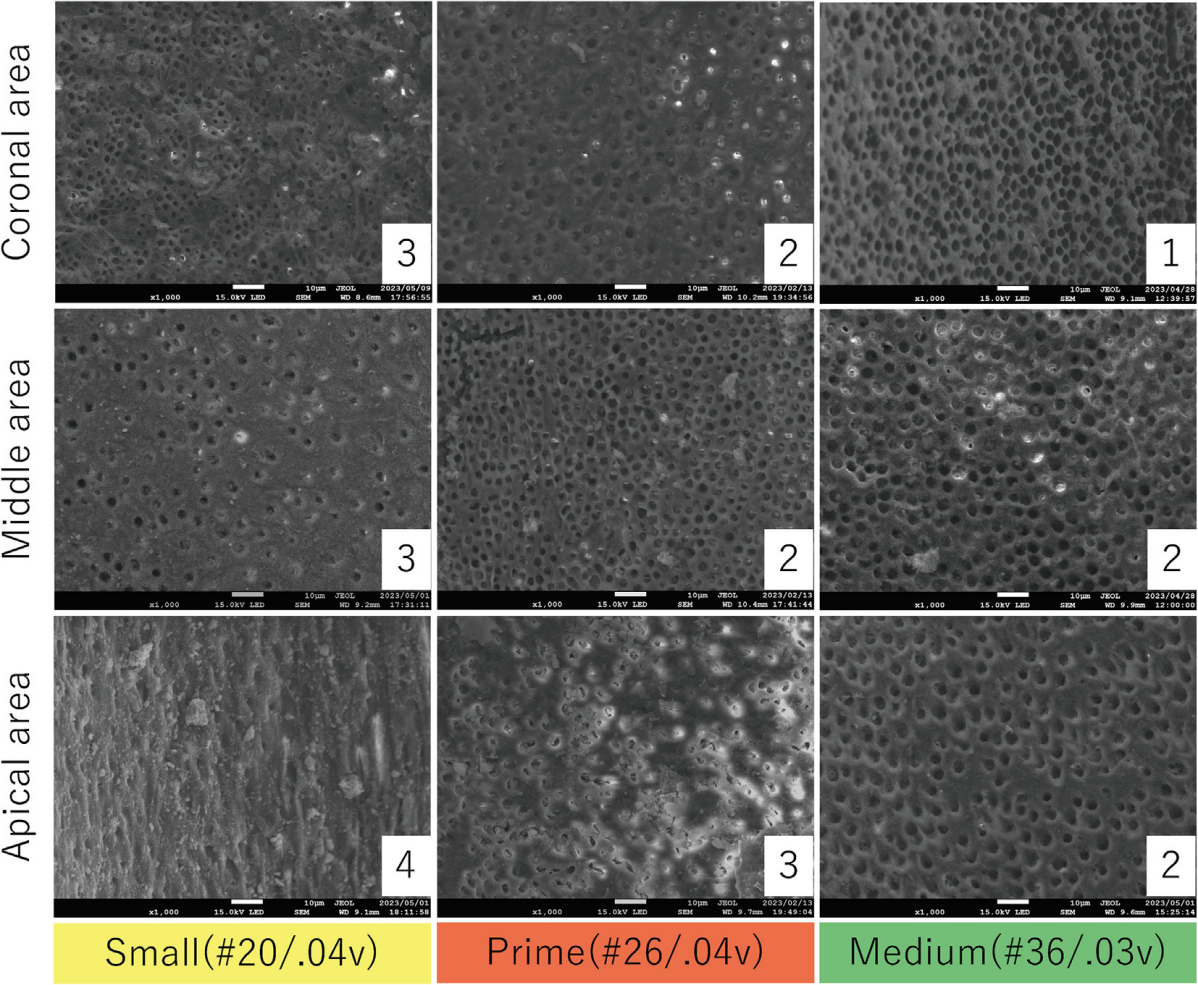
Representative scanning electron microscopy images showing the smear layer in Experiment 1. The scoring criteria are given in the legend of Fig. 1, and the score is shown in the lower right corner of each panel. Scale bar = 10 μm. Small, TruNatomy Shaping File Small; Prime, TruNatomy Shaping File Prime; Medium, TruNatomy Shaping File Medium.

**Figure 3:**
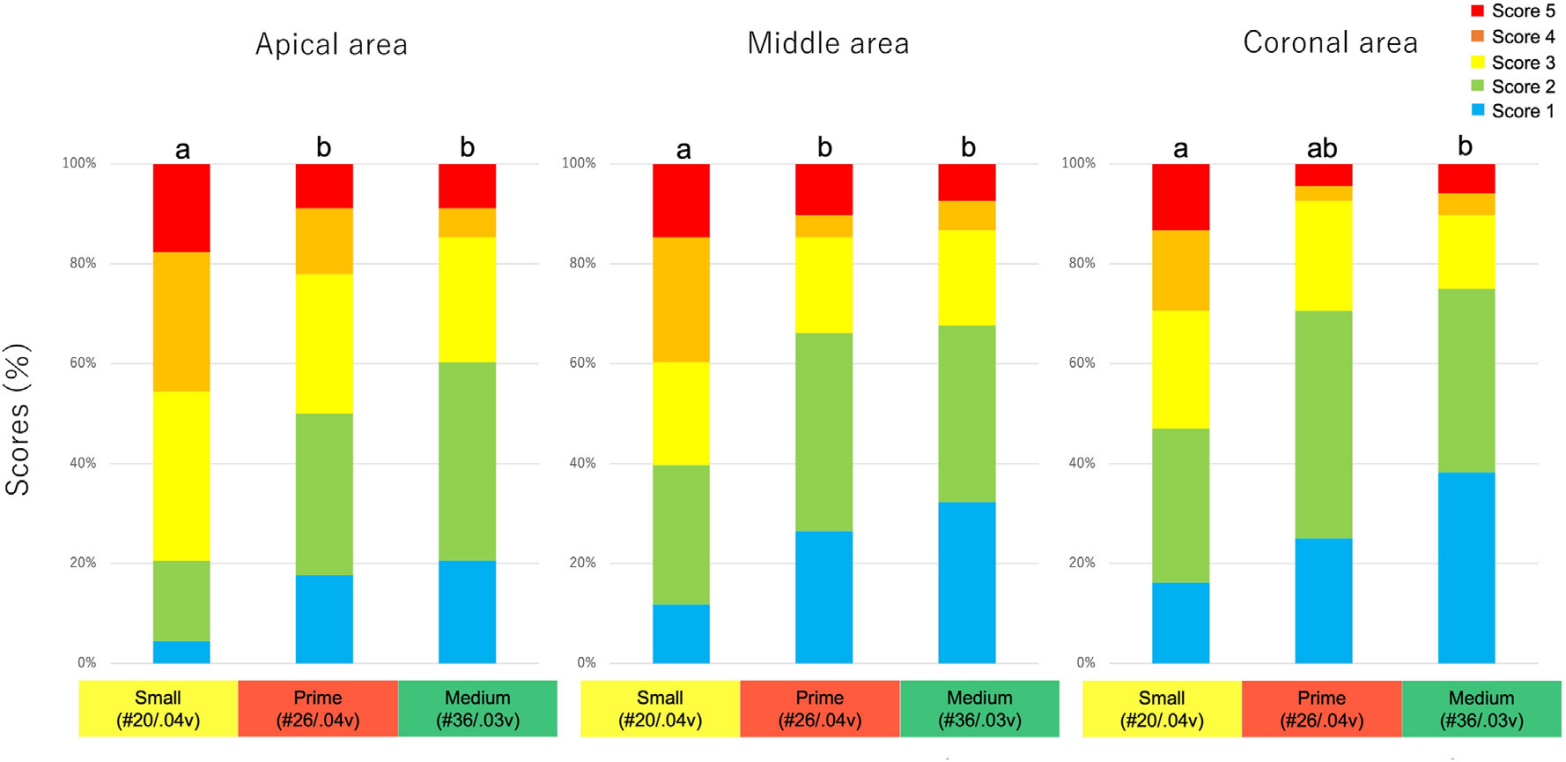
Percentage distribution of the smear layer score in Experiment 1. Root canals were instrumented up to TruNatomy Small, Prime, or Medium and irrigated using syringe irrigation. Different lowercase letters on each bar in an area indicate a significant difference between groups at *P* < 0.05. The scoring criteria are given in the legend of Fig. 1. Small, TruNatomy Shaping File Small; Prime, TruNatomy Shaping File Prime; Medium, TruNatomy Shaping File Medium.

As shown in Figure 2, Figure 3, the TruNatomy Small group showed significantly higher scores than the other groups in the apical and middle thirds of the canal (*P* < 0.05). In the coronal third, the scores for the TruNatomy Small group were significantly higher than those for the TruNatomy Medium group (*P* < 0.05).

### Experiment 2

**Figure 4:**
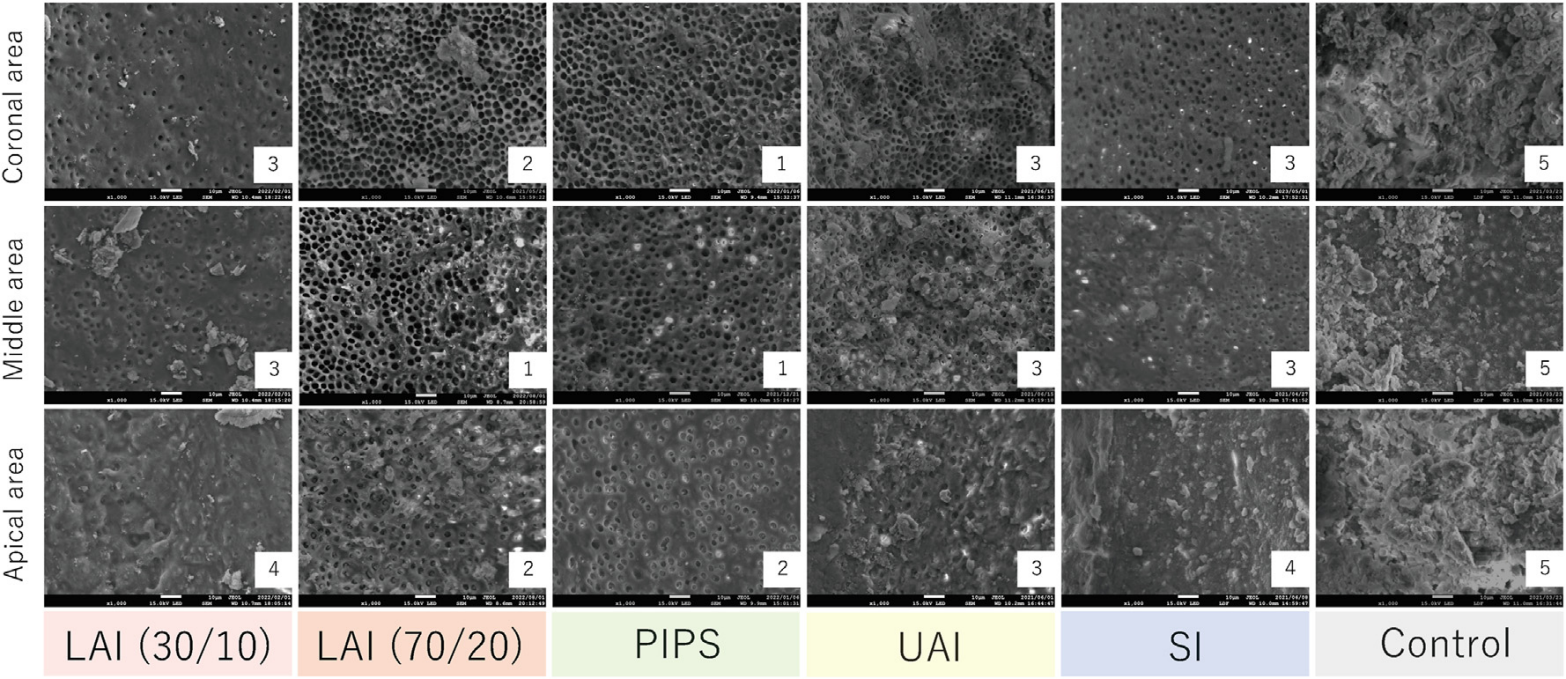
Representative scanning electron microscopy images showing the smear layer in Experiment 2. Root canals instrumented with TruNatomy Small and irrigated using different irrigation protocols. The scoring criteria are given in the legend of Fig. 1, and the score is shown in the lower right corner of each panel. Scale bar = 10 μm. LAI 30/10, laser-activated irrigation at 30 mJ, 10 Hz; LAI 70/20, laser-activated irrigation at 70 mJ, 20 Hz; PIPS, photon-induced photoacoustic streaming; UAI, ultrasonic-activated irrigation; SI, syringe irrigation; Control, no irrigation.

**Figure 5:**
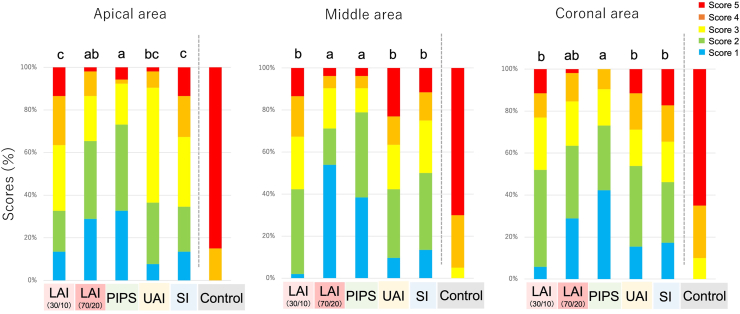
Percentage distribution of the smear layer score in Experiment 2. Root canals were instrumented with TruNatomy Small and irrigated using different irrigation protocols. Different lowercase letters on each bar in an area indicate a significant difference between groups at *P* < 0.05. The scoring criteria are given in Fig. 1. LAI 30/10, laser-activated irrigation at 30 mJ, 10 Hz; LAI 70/20, laser-activated irrigation at 70 mJ, 20 Hz; PIPS, photon-induced photoacoustic streaming; UAI, ultrasonic-activated irrigation; SI, syringe irrigation; Control, no irrigation.

As shown in Figure 4, Figure 5, the LAI 70/20 and PIPS groups showed significantly lower smear layer scores than the LAI 30/10 and SI groups in the apical third (*P* < 0.05) and than the other groups in the middle third (*P* < 0.05). The PIPS group showed significantly lower scores than the LAI 30/10, UAI, and SI groups in the coronal third (*P* < 0.05).

**Figure 6:**
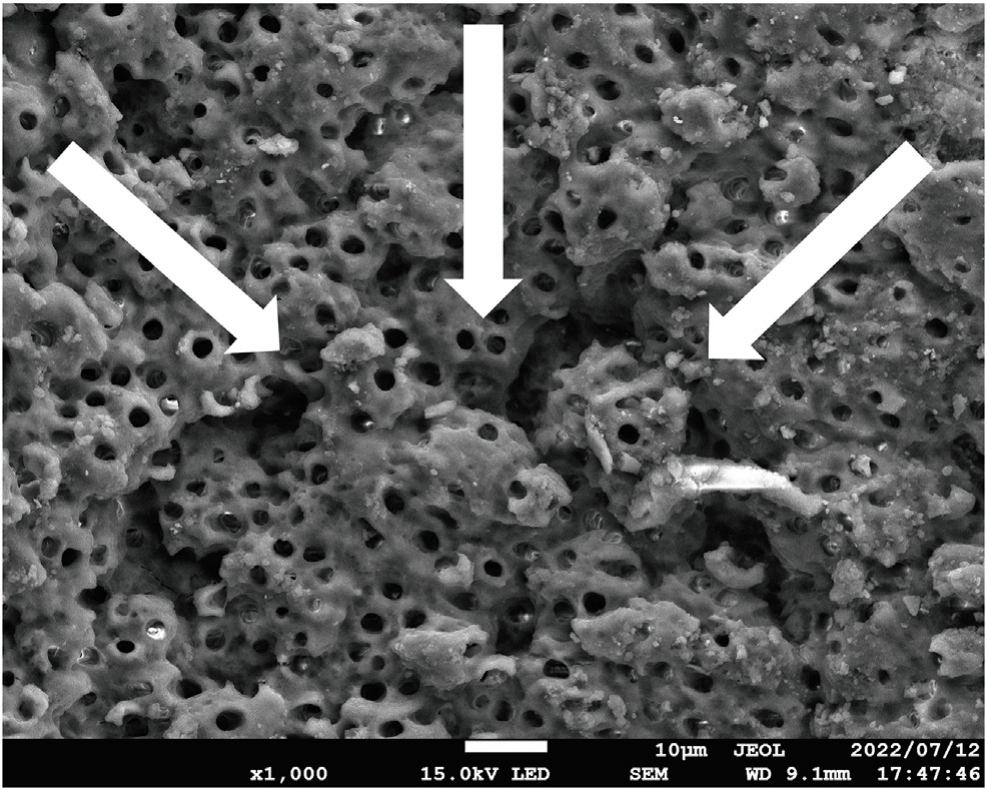
A representative scanning electron microscopy image from the middle area of the LAI 70/20 group, showing shallow crescent-shaped cavities (white arrows). Scale bar = 10 μm. LAI 70/20, laser-activated irrigation at 70 mJ, 20 Hz.

Seven out of 52 samples in the LAI 70/20 group exhibited shallow crescent-shaped cavities in the middle third (Fig. 6).

## Discussion

Although minimally invasive root canal instrumentation has increased in popularity, insufficient pathogen eradication is a concern, especially in infected root canals.^10^, ^11^, ^12^ It is crucial to consider that a more conservative approach to root canal instrumentation could potentially increase the risk of compromised cleaning efficacy. Moreover, only a few studies have examined the cleaning efficacy in conservatively instrumented root canals.^12^^,^^13^^,^^27^^,^^29^ The TruNatomy system is specifically designed to minimize unnecessary removal of root dentin,^8^^,^^9^ and has been reported to cause less apical transportation while preserving more dentin compared with several other nickel-titanium rotary and reciprocating systems.^30^^,^^31^ Therefore, in the present study, we used the TruNatomy system as a representative system for minimally invasive root canal instrumentation and compared the cleaning efficacy of different irrigation techniques. The smear layer removal efficacy was investigated considering that microorganisms are present in the smear layer and dentin tubules^32^^,^^33^ and that the smear layer prevents effective penetration of irrigants and may compromise the sealing ability of root filling materials.^34^^,^^35^ Our results demonstrated several significant differences in the smear layer scores between different instrumentation sizes and irrigation protocols. Thus, the null hypothesis was rejected.

Experiment 1 revealed that the cleaning efficacy of SI was significantly reduced when the final instrumentation was most conservatively performed with TruNatomy Small. This is consistent with the finding that more significant smear layer removal is achieved as the root canal instrumentation size increases.^12^ Based on these results, Experiment 2 aimed to determine the irrigation techniques that can improve the cleanliness of root canals instrumented with TruNatomy Small. Although the present experimental conditions were more conservative in terms of instrumentation size (#20/0.04 regressive taper), the findings support the recently reported efficiency of PIPS in canals instrumented to sizes #25/0.04 to 0.08 taper.^12^^,^^13^^,^^27^^,^^29^ Other studies have also demonstrated that PIPS is significantly more effective than ultrasonic techniques for root canals instrumented to a size #25/0.06 taper.^12^^,^^13^^,^^27^^,^^29^

In the present study, the PIPS group performed better than the LAI 30/10 group despite an identical power setting (0.3 W). This can be explained by the short pulse duration of PIPS (50 μs), which derives a high peak power inducing a “shockwave-like” effect that increases the pressure waves of irrigants.^24^^,^^25^ Thus, PIPS set at lower irradiation energy settings can have a higher cleaning efficacy than that of conventional LAI with a longer pulse duration.

In the PIPS group, the laser tip placed at the canal orifice may have allowed sufficient irrigant flow throughout the root canal. In the conventional LAI groups, however, the flow might have been compromised because of the volume of the laser tip placed in the narrow canals. Although conventional LAI may exert a certain cleaning effect even when applied at a distance,^21^, ^22^, ^23^^,^^36^ a higher irradiation power may be necessary for sufficient cleaning in the apical area of narrow root canals. In the LAI 70/20 group, SEM images demonstrated crescent-shaped cavities (Fig. 6). This finding suggested heat-induced injury to the canal wall, possibly due to the proximity of the laser tip to the root canal wall within the restricted canal space.^37^^,^^38^

SI and UAI exhibited comparable cleaning performances in all the root canal areas. UAI may be less effective in narrow root canals, where ultrasonic vibration is restricted. In the SI group, the ability of the fine, flexible needle used in this study to aid cleaning seemed limited; this is consistent with the finding that debridement is compromised with #20/0.04 or #25/0.04 instruments despite the use of narrow needles (30-G or 31-G).^13^ Thus, cleaning with SI and UAI may be inefficient in narrow canals, which might pose a negative impact on the clinical outcomes of root canal treatment due to insufficient pathogen eradication, especially in infected root canals.^10^, ^11^, ^12^

This study has several limitations. Firstly, SEM has the inherent limitation of not allowing longitudinal observations, although it is widely used for the evaluation of smear layers.^39^ Digital image analysis might be more objective and quantitative than the qualitative scoring system employed in the present study. Additionally, the use of teeth of unknown age might have influenced the results since the amount of sclerotic dentin may increase with age. Finally, the effect of canal curvature warrants further studies because cleaning the apical curvature may be more challenging. To assure the safety of irrigation procedures, future studies should be directed toward the apical extrusion of irrigants during each irrigation procedure.

In conclusion, conservative instrumentation using the TruNatomy Small reduced cleaning efficacy of syringe irrigation compared with instrumentation with TruNatomy Prime and Medium. In the conservatively instrumented canals, however, the PIPS technique, in which the laser tip is placed above the root canal orifice, demonstrated satisfactory smear layer removal without injury to the root canal wall.

## Declarations of competing interest

The authors declare no conflict of interest relevant to this study.

## References

[1] Siqueira J.F., Rôças I.N. (2008). Clinical implications and microbiology of bacterial persistence after treatment procedures. J Endod.

[2] Hülsmann M., Peters O.A., Dummer P.M.H. (2005). Mechanical preparation of root canals: shaping goals, techniques and means. Endod Top.

[3] Clark D., Khademi J. (2010). Modern molar endodontic access and directed dentin conservation. Dent Clin North Am.

[4] Gluskin A.H., Peters C.I., Peters O.A. (2014). Minimally invasive endodontics: challenging prevailing paradigms. Br Dent J.

[5] Neelakantan P., Vishwanath V., Taschieri S., Corbella S. (2022). Present status and future directions: minimally invasive root canal preparation and periradicular surgery. Int Endod J.

[6] Rundquist B.D., Versluis A. (2006). How does canal taper affect root stresses?. Int Endod J.

[7] Tang W., Wu Y., Smales R.J. (2010). Identifying and reducing risks for potential fractures in endodontically treated teeth. J Endod.

[8] Elnaghy A.M., Elsaka S.E., Mandorah A.O. (2020). In vitro comparison of cyclic fatigue resistance of TruNatomy in single and double curvature canals compared with different nickel-titanium rotary instruments. BMC Oral Health.

[9] Peters O.A., Arias A., Choi A. (2020). Mechanical properties of a novel nickel-titanium root canal instrument: stationary and dynamic tests. J Endod.

[10] Rollison S., Barnett F., Stevens R.H. (2002). Efficacy of bacterial removal from instrumented root canals in vitro related to instrumentation technique and size. Oral Surg Oral Med Oral Pathol Oral Radiol Endod.

[11] Khademi A., Yazdizadeh M., Feizianfard M. (2006). Determination of the minimum instrumentation size for penetration of irrigants to the apical third of root canal systems. J Endod.

[12] Plotino G., Özyürek T., Grande N.M., Gündoğar M. (2019). Influence of size and taper of basic root canal preparation on root canal cleanliness: a scanning electron microscopy study. Int Endod J.

[13] Boutsioukis C., Gutierrez Nova P. (2021). Syringe irrigation in minimally shaped root canals using 3 endodontic needles: a computational fluid dynamics study. J Endod.

[14] van der Sluis L.W., Versluis M., Wu M.K., Wesselink P.R. (2007). Passive ultrasonic irrigation of the root canal: a review of the literature. Int Endod J.

[15] Lee S.J., Wu M.K., Wesselink P.R. (2004). The effectiveness of syringe irrigation and ultrasonics to remove debris from simulated irregularities within prepared root canal walls. Int Endod J.

[16] Blanken J., De Moor R.J., Meire M., Verdaasdonk R. (2009). Laser induced explosive vapor and cavitation resulting in effective irrigation of the root canal. Part 1: a visualization study. Laser Surg Med.

[17] de Groot S.D., Verhaagen B., Versluis M., Wu M.K., Wesselink P.R., van der Sluis L.W. (2009). Laser-activated irrigation within root canals: cleaning efficacy and flow visualization. Int Endod J.

[18] De Moor R.J., Meire M., Goharkhay K., Moritz A., Vanobbergen J. (2010). Efficacy of ultrasonic versus laser-activated irrigation to remove artificially placed dentin debris plugs. J Endod.

[19] Lloyd A., Uhles J.P., Clement D.J., Garcia-Godoy F. (2014). Elimination of intracanal tissue and debris through a novel laser-activated system assessed using high-resolution micro-computed tomography: a pilot study. J Endod.

[20] Swimberghe R.C.D., De Clercq A., De Moor R.J.G., Meire M.A. (2019). Efficacy of sonically, ultrasonically and laser-activated irrigation in removing a biofilm-mimicking hydrogel from an isthmus model. Int Endod J.

[21] Aung N.P.S., Watanabe S., Okiji T. (2021). Er:YAG laser-activated irrigation in comparison with different irrigation systems for cleaning the apical root canal area beyond ledge. Photobiomodul Photomed Laser Surg.

[22] Hoshihara Y., Watanabe S., Kouno A., Yao K., Okiji T. (2021). Effect of tip insertion depth and irradiation parameters on the efficacy of cleaning calcium hydroxide from simulated lateral canals using Er:YAG laser- or ultrasonic-activated irrigation. J Dent Sci.

[23] Liu J., Watanabe S., Mochizuki S., Kouno A., Okiji T. (2023). Comparison of vapor bubble kinetics and cleaning efficacy of different root canal irrigation techniques in the apical area beyond the fractured instrument. J Dent Sci.

[24] DiVito E., Peters O.A., Olivi G. (2012). Effectiveness of the erbium:YAG laser and new design radial and stripped tips in removing the smear layer after root canal instrumentation. Laser Med Sci.

[25] Peters O.A., Bardsley S., Fong J., Pandher G., Divito E. (2011). Disinfection of root canals with photon-initiated photoacoustic streaming. J Endod.

[26] Schneider S.W. (1971). A comparison of canal preparations in straight and curved root canals. Oral Surg Oral Med Oral Pathol.

[27] Mancini M., Cerroni L., Palopoli P. (2021). FESEM evaluation of smear layer removal from conservatively shaped canals: laser activated irrigation (PIPS and SWEEPS) compared to sonic and passive ultrasonic activation-an ex vivo study. BMC Oral Health.

[28] Hülsmann M., Rümmelin C., Schäfers F. (1997). Root canal cleanliness after preparation with different endodontic handpieces and hand instruments: a comparative SEM investigation. J Endod.

[29] Eldeeb I.M., Nawar N.N., Saber S.M., Hassanein E.E., Schäfer E. (2021). Smear layer removal and sealer penetration with different tapers after using photon-initiated photoacoustic streaming technique. Clin Oral Invest.

[30] Kim H., Jeon S.J., Seo M.S. (2021). Comparison of the canal transportation of ProTaper GOLD, WaveOne GOLD, and TruNatomy in simulated double-curved canals. BMC Oral Health.

[31] Pérez Morales M.L.N., González Sánchez J.A., Olivieri J.G. (2021). Micro-computed tomographic assessment and comparative study of the shaping ability of 6 nickel-titanium files: an in vitro study. J Endod.

[32] Baker N.A., Eleazer P.D., Averbach R.E., Seltzer S. (1975). Scanning electron microscopic study of the efficacy of various irrigating solutions. J Endod.

[33] Yamada R.S., Armas A., Goldman M., Lin P.S. (1983). A scanning electron microscopic comparison of a high volume final flush with several irrigating solutions: Part 3. J Endod.

[34] White R.R., Goldman M., Lin P.S. (1984). The influence of the smeared layer upon dentinal tubule penetration by plastic filling materials. J Endod.

[35] Kennedy W.A., Walker W.A., Gough R.W. (1986). Smear layer removal effects on apical leakage. J Endod.

[36] Kouno A., Watanabe S., Hongo T., Yao K., Satake K., Okiji T. (2020). Effect of pulse energy, pulse frequency, and tip diameter on intracanal vaporized bubble kinetics and apical pressure during laser-activated irrigation using Er:YAG laser. Photobiomodul Photomed Laser Surg.

[37] Kokuzawa C., Ebihara A., Watanabe S. (2012). Shaping of the root canal using Er:YAG laser irradiation. Photomed Laser Surg.

[38] Watanabe S., Saegusa H., Anjo T., Ebihara A., Kobayashi C., Suda H. (2010). Dentin strain induced by laser irradiation. Aust Endod J.

[39] De-Deus G., Reis C., Paciornik S. (2011). Critical appraisal of published smear layer-removal studies: methodological issues. Oral Surg Oral Med Oral Pathol Oral Radiol Endod.

